# Inhibition of triple-negative breast cancer growth via delphinidin-mediated suppression of the JAK2/STAT3/PD-L1 pathway

**DOI:** 10.29219/fnr.v68.10974

**Published:** 2024-12-31

**Authors:** Xiaoping Yu, Xiaolong Song, Jiali Yan, Ziting Xiong, Lujie Zheng, Yan Luo, Fengcheng Deng, Yanfeng Zhu

**Affiliations:** 1 School of Medicine and Nursing, Chengdu University, Chengdu, China; 2 School of Public Health, Chengdu Medical College, Chengdu, China; 3 Thyroid Breast Surgery, Chengdu Seventh People’s Hospital, Tumor Affiliated Hospital of Chengdu Medical College, Chengdu, China

**Keywords:** delphinidin, TNBC, JAK2/STAT3, PD-L1, exosome, phytochemicals

## Abstract

**Background:**

Breast cancer is a leading cause of cancer-related mortality among women globally, with triple-negative breast cancer (TNBC) being particularly aggressive. Delphinidin (Dp), an anthocyanin monomer, has shown promising health benefits.

**Objective:**

This study investigates the effects of Dp on TNBC and aims to elucidate its specific mechanisms of action.

**Design:**

We utilized cell counting kit-8 (CCK-8) assays, colony formation assays, and scratch assays to evaluate the influence of Dp on the proliferation and migration of TNBC cells. Flow cytometry was employed to analyze programmed cell death-ligand 1 (PD-L1) and Cluster of Differentiation 69 expression, while Western blotting assessed the levels of PD-L1, Janus Kinase 2 (JAK2), Signal Transducer and Activator of Transcription 3 (STAT3), p-JAK2, p-STAT3, and exosomal marker proteins. Additionally, enzyme-linked immunosorbent assay (ELISA) was conducted to measure concentrations of PD-L1, interferon-γ (IFN-γ), and tumor necrosis factor-β (TNF-β).

**Results:**

Dp effectively inhibited TNBC cell proliferation and migration, as evidenced by CCK-8, colony formation, and scratch assays. Flow cytometry and Western blot analysis indicated a reduction in PD-L1 expression in TNBC cells. Meanwhile, we successfully isolated TNBC cell-derived exosomes, with ELISA experiments showing a decrease in PD-L1 expression in these exosomes following Dp treatment. In a co-culture system with TNBC and Jurkat cells, Dp enhanced Cluster of Differentiation 69 expression and reactivated Jurkat cells, resulting in increased secretion of IFN-γ and TNF-β. Additionally, Dp significantly reduced the p-JAK2/JAK2 and p-STAT3/STAT3 ratios in TNBC cells.

**Conclusion:**

Dp may exert its anti-TNBC effects by downregulating PD-L1 expression in TNBC cells and exosomes through the JAK2/STAT3 signaling pathway, potentially restoring T cell activity and modifying the tumor microenvironment.

## Popular scientific summary

- Delphinidin inhibits the proliferation and migration of triple-negative breast cancer cells.
- Delphinidin decreases PD-L1 expression in triple-negative breast cancer cells and their exosomes and restores T-cell activity.
- Delphinidin may modulate the JAK2/STAT3 pathway to achieve PD-L1 downregulation.

The ‘2020 Global Cancer Burden Report’ by the International Agency for Research on Cancer, a branch of the World Health Organization, highlighted breast cancer as the most frequently diagnosed cancer among women worldwide, surpassing lung cancer (1). Triple-negative breast cancer (TNBC) represents approximately 15% of all breast cancer cases and is defined by the absence of estrogen receptors (ER), progesterone receptors (PR), and human epidermal growth factor receptor-2 (HER-2) (2, 3). This subtype is particularly aggressive, characterized by high malignancy, early recurrence, a high incidence of visceral metastasis, and significant treatment challenges (4). While, chemotherapy remains the primary treatment strategy for TNBC, conventional broad-spectrum chemotherapeutic agents can also damage normal cells and cause substantial side effects.

Immunotherapy has recently emerged as a crucial advancement in cancer treatment, often regarded as the fourth modality following surgery, radiotherapy, and chemotherapy. Research suggests that 50–80% of TNBC cases exhibit programmed cell death-ligand 1 (PD-L1) positivity, a key factor in immune evasion that correlates with immune cells infiltration (5). PD-L1 is expressed not only on the surface of tumor cells but also on exosomes they secrete. This expression plays an important role in modulating anti-tumor immune responses (6). Exosomes are intrinsically linked to the development and progression of various diseases. It is well-established that cancer cell-derived exosomes can stimulate cancer growth and migration and influence immune cell responses, thus promoting cancer progression and metastasis (7). Studies have shown that PD-L1 on the surface of tumor-derived exosomes can suppress anti-tumor immunity locally or systemically by binding to programmed death-1 (PD-1) on T cells, facilitating immune escape and promoting tumor progression (8). Further investigations into the regulation of PD-L1 levels across various tumors revealed that cancer cells predominantly secrete PD-L1 via exosomes rather than display it on their surface (9). Therefore, targeting PD-L1 expression on exosomes presents a promising therapeutic approach to counteract tumor immune evasion.

Anthocyanins, naturally occurring compounds in plants, offer significant health benefits. Among these, delphinidin (Dp) is particularly renowned for its potent biological effects, attributed to the three hydroxyl groups on its benzene ring. This unique structure enhances its anticancer properties, surpassing the efficacy of other anthocyanins (10, 11). Recent studies, such as those by Mazewski C and colleagues (12), have demonstrated that Dp induces apoptosis in tumor cells and decreases PD-L1 protein expression in colorectal cancer HCT-116 cells. Furthermore, Dp has been shown to reduce PD-1 expression in human peripheral blood mononuclear cells within a tumor microenvironment (TME) model. This suggests that Dp may inhibit colorectal cancer progression by blocking the PD-L1/PD-1 pathway. However, the precise mechanism by which Dp modulates PD-L1 to prevent immune escape in TNBC remains to be clarified.

The PI3K/AKT and Janus Kinase 2 (JAK2)/Signal Transducer and Activator of Transcription 3 (STAT3) signaling pathways are pivotal in regulating downstream transcription factors’ activation and nuclear translocation, thereby enhancing PD-L1 transcription (13). The JAK2/STAT3 pathway, consisting of tyrosine kinase-associated receptors, the tyrosine kinase JAK2, and the transcription factor STAT3, plays an essential role in cell proliferation and differentiation (14). Its activation is critical for establishing a tumor’s inflammatory microenvironment and is closely associated with the onset and progression of many human cancers. Additionally, Dp has also been shown to regulate the JAK2/STAT3 signaling pathway, inducing apoptosis in colorectal cancer cells (15).

This study aims to explore the mechanisms by which Dp targets PD-L1 to prevent immune escape or attenuate immune suppression within the TME. By investigating how Dp reduces PD-L1 expression, we seek to provide a scientific basis for understanding the role of phytochemicals in impeding immune escape and combating tumors. Our findings may offer theoretical support for developing dietary supplements for the chemoprevention of TNBC.

## Materials and methods

### Reagents

All chemicals used in this study were of analytical reagent grade. Dp was sourced from Sigma-Aldrich (Purity ≥ 95%, Cat# 43725), and Atezolizumab (Anti-PD-L1) was obtained from MedChemExpress (Purity ≥ 98%, Cat# HY-P9904). Dulbecco’s Modified Eagle Medium (DMEM Cat# SH30285) and RPMI-1640 medium (Cat# SH30255), along with 1% penicillin/streptomycin solution (Cat# SV30010) were purchased from HyClone. Trypsin and fetal bovine serum were acquired from Gibco (USA). Phorbol myristate acetate (PMA, Cat# S1819) and ionomycin (Ion, Cat# S1672) were procured from Beyotime Biotechnology.

### Cell culture

Human TNBC cell lines MDA-MB-231 (RRID: CVCL_0062) and BT-549 (RRID: CVCL_10920), the normal mammary epithelial cell line MCF-10A (RRID: CVCL_0598), and Jurkat cells (RRID: CVCL_0065) were acquired from the Cell Bank of the Shanghai Institutes for Biological Sciences, Chinese Academy of Sciences. MDA-MB-231 cells were cultured in DMEM supplemented with 10% fetal bovine serum and 1% penicillin/streptomycin. BT-549 and Jurkat cells were maintained in RPMI-1640 medium with 10% fetal bovine serum and 1% penicillin/streptomycin. MCF-10A cells were grown in DMEM medium containing 5% horse serum, 0.5 μg/ml hydrocortisone, 10 μg/ml insulin, 1% non-essential amino acids, and 1% penicillin/streptomycin. All cultures were kept at 37°C in a humidified atmosphere with 5% CO_2_. Cells were subcultured upon reaching 80–90% confluence using trypsin digestion.

### Cell viability assay

Cells in the logarithmic growth phase from MDA-MB-231, BT-549, and MCF-10A lines were seeded into a 96-well plate at a density of approximately 5 × 10^3^ cells/well. Wells designated for Dp treatment were exposed to a gradient of Dp concentrations, with Dimethyl sulfoxide (DMSO) serving as the solvent control. Each category was tested in quintuplicate, and the experiment was conducted with at least three biological replicates. After 48 h of treatment, 100 μL of complete culture medium supplemented with 10 μL of Cell Counting Kit-8 (CCK-8) (Biosharp, Cat# BS350) was added to each well. The plates were then incubated for 30 min in a culture incubator. Cell viability was determined by measuring absorbance at 450 nm using a microplate reader.

### Colony formation assay

TNBC cells in their logarithmic growth phase were seeded at a density of 5 × 10^3^ cells/well in a 6-well plate, with three biological replicates conducted. After seeding, the cells were transferred to an incubator to stabilize. They were then treated with varying concentrations of Dp for 14 days. Following treatment, cells were fixed with 4% paraformaldehyde and stained with 0.1% crystal violet. Post-staining, the cells were rinsed with Phosphate buffered saline (PBS). Images were captured using an inverted microscope, and colony numbers were quantified with ImageJ software.

### Wound healing assay

TNBC cells were seeded at a density of 7 × 10^5^ cells/well in a 6-well plate, with three biological replicates performed. Upon reaching 80–90% confluence, a scratch was made using a 10 μL pipette tip guided by a ruler. Wells were then washed with PBS to remove detached cells. Subsequently, the cells were treated with Dp, and scratch closure was recorded for each group at 0, 24, 48, and 72 h. The scratch closure was analyzed using ImageJ software.

### Western blot analysis

After Dp treatment, TNBC cells were collected and lysed in RIPA buffer (Beyotime, Cat# P0013B) with protease and phosphatase inhibitors to extract total cellular proteins. Protein concentrations were determined using the BCA Protein Assay Kit (Beyotime, Cat# P0012). For each sample, 15–30 μg of protein were separated by SDS-PAGE and transferred onto a PVDF membrane (Millipore, Cat# ISEQ85R). The membrane was blocked with 5% BSA at room temperature for 1 h, followed by overnight incubation at 4°C with specific primary antibodies. After washing with Tris Buffered Saline with Tween-20 (TBST) (Biosharp, Cat# BL315B), the membrane was incubated with HRP-conjugated secondary antibodies at room temperature for 1 h. Visualization was accomplished using an ECL solution (Adamas Life, Cat# E8059) and a chemiluminescence detection system. The following primary antibodies and dilutions were used: PD-L1 (Proteintech Cat# 66248-1-Ig, RRID: AB_2756526, 1:2000), PD-L1 (Cell Signaling Technology Cat# 60475, RRID: AB_2924680, 1:1000), JAK2 (Affinity Biosciences Cat# AF6022, RRID: AB_2834956, 1:2000), p-JAK2 (Affinity Biosciences Cat# AF3024, RRID: AB_2834455, 1:2000), STAT3 (Affinity Biosciences Cat# AF6294, RRID: AB_2835144, 1:2000), p-STAT3 (Affinity Biosciences Cat# AF3293, RRID: AB_2810278, 1:2000), Tumor Susceptibility Gene 101 (Proteintech Cat# 28283-1-AP, RRID:AB_2881104, 1:2000), ALG-2-interacting protein X (Proteintech Cat# 12422-1-AP, RRID: AB_2162467, 1:5000), Cluster of Differentiation 9 (Proteintech Cat# 20597-1-AP, RRID: AB_2878706, 1:1000), GAPDH (ServiceBio Cat# GB15004-100, RRID: AB_2943040, 1:3000). The secondary antibody used was Anti-rabbit IgG, HRP-linked Antibody (Cell Signaling Technology Cat# 7074, RRID: AB_2099233, 1:3000).

### Enzyme-linked immunosorbent assay analysis

PD-L1, Tumor Necrosis Factor-β (TNF-β), and Interferon-γ (INF-γ) levels in mouse serum and cell culture media were measured using Enzyme-Linked Immunosorbent Assay (ELISA) kits, following the manufacture’s instructions. Concentrations were determined by referencing standard curves.

### Analysis of PD-L1 on the Surface of TNBC Cells by Flow Cytometry

MDA-MB-231 and BT-549 cells were washed with pre-chilled PBS containing 1% BSA. They were then centrifuged at 400 g for 5 min at 4°C. After discarding the supernatant, cells were resuspended in 100 μL of PBS/BSA solution containing a PD-L1 antibody (Thermo Fisher Scientific Cat# 47-5983-41, RRID: AB_2688258) for flow cytometry. Cells were incubated in the dark at 4°C for 30 min, followed by washing with PBS/BSA solution under light-protected conditions and centrifugation. The cell pellet was resuspended in 200 μL of PBS/BSA solution., and PD-L1 expression was assessed using a flow cytometer.

### Exosome Isolation and Characterization

Exosomes were isolated from the supernatant of TNBC cells through a series of centrifugation steps designed to remove cellular debris and vesicles. Following centrifugation, the clarified supernatant was filtered through a 0.22 μm filter to eliminate any remaining particulate matter. The filtrate was then ultracentrifuged at 100,000 g for 70 min at 4°C to pellet the exosomes. This pellet was resuspended in precooled PBS and subjected to another rounds of ultracentrifuged under the same conditions to purify the exosome preparations. The isolated exosomes were resuspended in precooled PBS and stored at −80°C until further analysis. The expression of exosomal marker proteins, including ALG-2-interacting protein X, Cluster of Differentiation 9, and Tumor Susceptibility Gene 101, was confirmed via Western blotting. Exosome morphology was assessed using transmission electron microscopy (TEM), which revealed their characteristic cup-shaped structure. Nanoparticle tracking analysis (NTA) was also conducted to determine the size distribution of exosomes, providing essential data on their dimensional properties and concentration.

### Determination of Jurkat cells activation by flow cytometry

Jurkat cells with robust proliferation were seeded at a density of 2 × 10^4^ cells/well in a 24-well plate. Various concentrations of a PMA and Ion mixture were added to initiate activation, and the cells were incubated at 37°C for 4.5 h. Flow cytometric analysis was conducted according to protocol 2.11, using the expression level of PD-1 (Thermo Fisher Scientific Cat# 11-9985-85, RRID: AB_465473) as a marker to establish the optimal concentrations of PMA and Ion for subsequent experiments.

### Cluster of differentiation 69 analysis in a co-culture system by flow cytometry

TNBC cells in the logarithmic growth phase were seeded at 4 × 10^4^ cells/well in the upper chamber of a 24-well Transwell culture plate, while Jurkat cells were seeded at 4 × 10^5^ cells/well in the lower chamber. For the control group, Jurkat cells were activated with PMA and Ion and seeded at 4 × 10^5^ cells/well in the lower chamber. After 24, 48, and 72 h of culture, Jurkat cells from the lower chamber were collected and centrifuged at 400 g for 5 min at 4°C. The supernatant was used to measure the cytokines IFN-γ and TNF-β, while the cell pellet was analyzed by flow cytometry to assess the expression of Cluster of Differentiation 69 (Bioss Cat# bs-2499R-PE-Cy7, RRID: AB_11093790).

### Molecular docking

Compound structure files were sourced from the PubChem database (https://pubchem.ncbi.nlm.nih.gov/) and converted from SDF to PDB format using Open Babel 2.3.2 software. Receptor proteins were obtained from the PDB database. Using PYMOL 2.3.4, water molecules and ligands were removed from receptor proteins, which were then imported into AutoDockTools. Here, hydrogen atoms were added, charges calculated, and the structures were prepared for docking. Both receptor proteins and ligand molecules were converted to Protein Data Bank Quantification Toolkit format. Molecular docking was performed using AutoDock Vina 1.1.2, and results were visualized with PYMOL.

### Molecular dynamics simulation

Molecular dynamics (MD) simulations were performed using GROMACS2020.3 to investigate protein-ligand interactions (16, 17). The amber99sb-ildn and the general amber force field (GAFF) force fields were used for proteins and ligands, respectively. The simulation box ensured a minimum 1.0 nm distance from the protein atoms and was filled with SPC216 water molecules, neutralized with Na^+^ and Cl^−^ ions. The system was optimized using the steepest descent method. NVT and NPT ensembles were run for 100 ps at 300 K and 1 bar for pre-equilibration. A subsequent 100 ns MD simulation was performed, with V-rescale used for temperature control and Parrinello-Rahman for pressure control (18). Leapfrog integration with a 2 fs timestep solved the Newton equations, using PME for electrostatics and LINCS for bond constraints. Visualization and trajectory analysis were conducted using Visual Molecular Dynamics (VMD) version 1.9.3 and PyMOL version 2.4.1 (19), Binding free energy was calculated using gmx_mmpbsa (http://jerkwin.github.io/gmxtool).

### Statistical analysis

Data are presented as mean ± standard error of the mean (SEM). Statistical analyses were conducted using GraphPad Prism 9.0 and SPSS 25.0. Differences between groups were assessed using the *t*-test and one-way ANOVA. A *p*-value of < 0.05 was considered statistically significant, while a *p*-value of < 0.01 was deemed highly significant, where * denotes *p* < 0.05, ** denotes *p* < 0.01.

## Results

### Dp suppresses the proliferation of TNBC cells

**Fig. 1:**
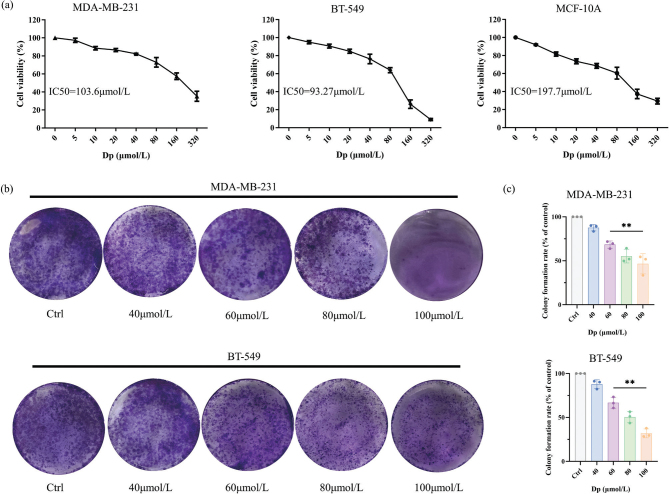
Delphinidin (Dp) suppresses the proliferation of triple-negative breast cancer (TNBC) cells. (a) The cell counting kit-8 (CCK-8) assay was used to assess the impact of Dp on the viability of MDA-MB-231, BT-549, and MCF-10A cells. (b) Colony formation assays were conducted to evaluate the effects of Dp on the proliferative capabilities of MDA-MB-231 and BT-549 cells. (c) Analysis of the clonogenic assay results using ImageJ software. ** indicates *p* < 0.01, *vs.* control group.

To evaluate the cytotoxic effects of Dp on TNBC and normal mammary epithelial cells, MDA-MB-231, BT-549, and MCF-10A cells were treated with varying concentrations of Dp for 48 h. The CCK-8 assay results demonstrated that Dp inhibited TNBC cell proliferation in a dose-dependent manner. The calculated IC50 values were 103.6 μmol/L for MDA-MB-231 cells, 93.27 μmol/L for BT-549 cells, and 197.7 μmol/L for MCF-10A cells (Fig. 1a). Further analysis using colony formation assays demonstrated that Dp significantly suppressed the proliferative potential of TNBC cells at concentrations ranging from 60 to 100 μmol/L (Fig. 1b and c). In conclusion, Dp exerts cytotoxic effects on TNBC cells, effectively inhibiting their proliferation.

### Dp impairs the migration of TNBC cells

**Fig. 2:**
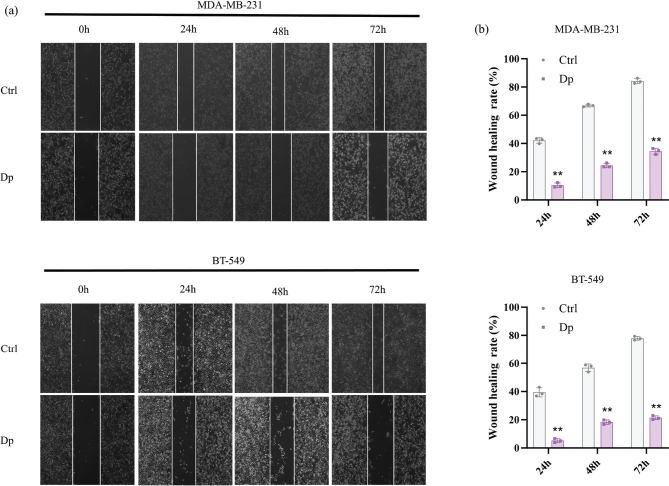
Delphinidin (Dp) inhibits triple-negative breast cancer (TNBC) cells’ migratory capabilities. (a) Wound healing assays were performed to determine the effects of 80 μmol/L Dp on the migration of TNBC cells. (b) Analysis of the scratch healing results using ImageJ software showed that 80 μmol/L Dp reduced the wound closure rates of TNBC cells at different treatment times (24, 48, and 72 h). ** indicates *p* < 0.01, *vs.* control group.

TNBC cells are known for their strong invasive and migratory capabilities. To evaluate the effect of Dp on these properties, wound healing assays were conducted over 24, 48, and 72 h. The results showed that Dp progressively inhibited the migration of MDA-MB-231 and BT-549 cells overtime (Fig. 2a). Treatment with 80 μmol/L Dp significantly reduced cell migration compared to the control group at each time point. The differences in wound closure rates between the treated and control groups were highly significant (Fig. 2b). These findings indicate that Dp effectively limits the motility and substantially impairs the migratory capacity of MDA-MB-231 and BT-549 cells.

### Dp reduces PD-L1 expression in TNBC cells

**Fig. 3:**
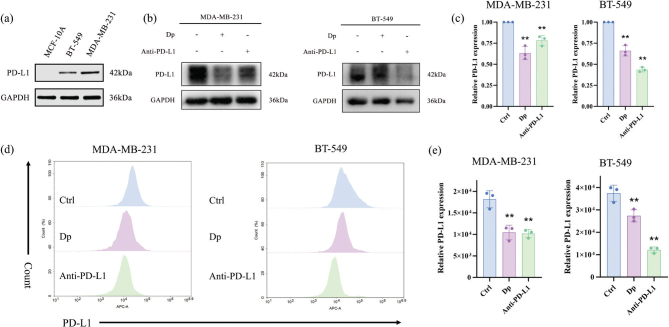
Delphinidin (Dp) reduces programmed cell death-ligand 1 (PD-L1) expression in triple-negative breast cancer (TNBC) cells. (a) Western blot assessed PD-L1 expression in MDA-MB-231, BT-549, and MCF-10A cells. (b, c) Treatment of TNBC cells with 80 μmol/L Dp and 30 μg/L Atezolizumab for 48 h resulted in a decrease in PD-L1 expression, as demonstrated by Western blot analysis, with grayscale analysis performed using ImageJ software. (d, e) Flow cytometry revealed a reduction in PD-L1 expression on the cell membranes of TNBC cells following treatment with 80 μmol/L Dp and 30 μg/L Atezolizumab. ** indicates *p* < 0.01, * indicates *p* < 0.05, *vs.* control group.

The expression of PD-L1 in normal mammary epithelial cells and TNBC cells was investigated. Western blot analysis revealed low PD-L1 expression in normal MCF-10A cells, whereas it was significantly higher in TNBC cells, with MDA-MB-231 cells showing the highest levels (Fig. 3a). MDA-MB-231 and BT-549 cells were subsequently treated with Dp and Anti-PD-L1 for 48 h. The western blot results demonstrated that Dp significantly decreased PD-L1 expression in TNBC cells (Fig. 3b and c). This reduction was confirmed by flow cytometry analysis, which showed a consistent decrease in surface PD-L1 expression on TNBC cells (Fig. 3d and e). These findings suggest that Dp and Anti-PD-L1 have similar effects in reducing PD-L1 expression, indicating that Dp effectively diminishes PD-L1 levels in TNBC cells.

### Dp decreases PD-L1 expression in exosomes derived from TNBC cells

**Fig. 4:**
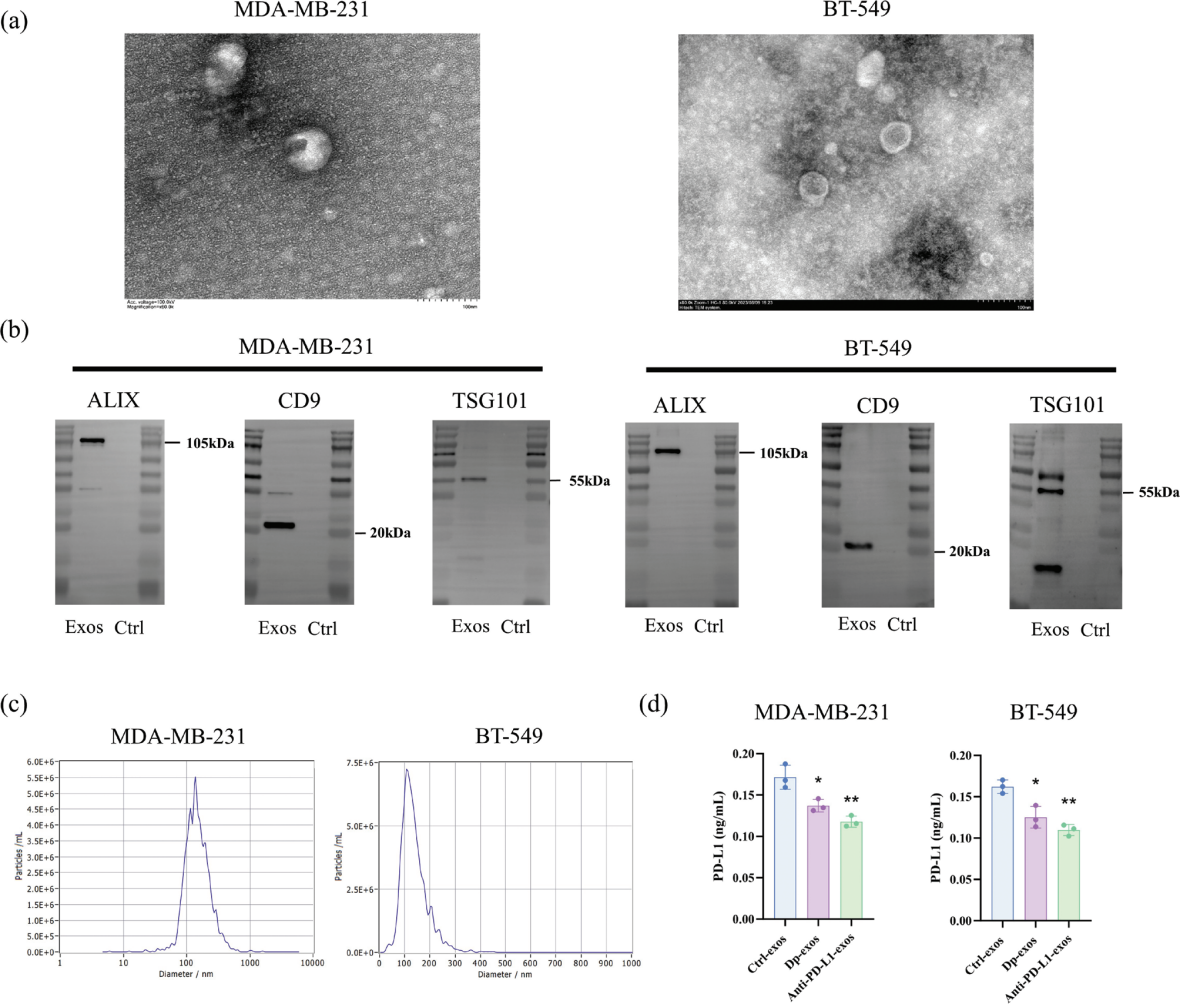
Morphology, size analysis, and marker protein expression of exosomes derived from triple-negative breast cancer (TNBC) cells, with delphinidin (Dp) reducing programmed cell death-ligand 1 (PD-L1) expression in exosomes from TNBC cells. (a) Transmission electron microscopy (TEM) revealed the morphology of exosomes isolated from the culture medium of TNBC cells, scale bar 100 nm. (b) Western blot analysis identified exosomal marker proteins ALG-2-interacting protein X, Cluster of Differentiation 9, and Tumor Susceptibility Gene 101 expression in exosomes from TNBC cells, where exosome samples are labeled as Exos, and the control group represents the cell culture medium without exosomes. (c) Nanoparticle tracking analysis (NTA) determined the size of exosomes derived from MDA-MB-231 and BT-549. (d) Enzyme-Linked Immunosorbent Assay (ELISA) kit assessed PD-L1 expression in TNBC-derived exosomes. ** indicates *p* < 0.01, * indicates *p* < 0.05 *vs.* control group.

Exosomes from cancer cells play a crucial role in cancer development and progression. We isolated exosomes and characterized exosomes from the culture medium of TNBC cells. TEM revealed that TNBC-derived exosomes exhibited a typical ‘cup-shaped’ morphology with clear, rounded, or oval bilayer structures (Fig. 4a). Western blot analysis identified specific exosomal marker proteins ALG-2-interacting protein X, Cluster of Differentiation 9, and Tumor Susceptibility Gene 101 (Fig. 4b). NTA showed that exosomes from MDA-MB-231 cells had an average diameter of 163.5 nm, while those from BT-549 cells measured 111.7 nm (Fig. 4c). To determine if Dp reduces PD-L1 expression in exosomes, we measured PD-L1 levels using an ELISA kit post-Dp treatment. The results indicated a decrease in PD-L1 expression in exosomes from Dp-treated TNBC cells (Fig. 4d). These findings confirm the successful isolation of exosomes from TNBC cells and demonstrate that Dp effectively reduces PD-L1 expression in these exosomes.

### Dp reduces exosomal PD-L1 expression and reactivates Jurkat cells

**Fig. 5:**
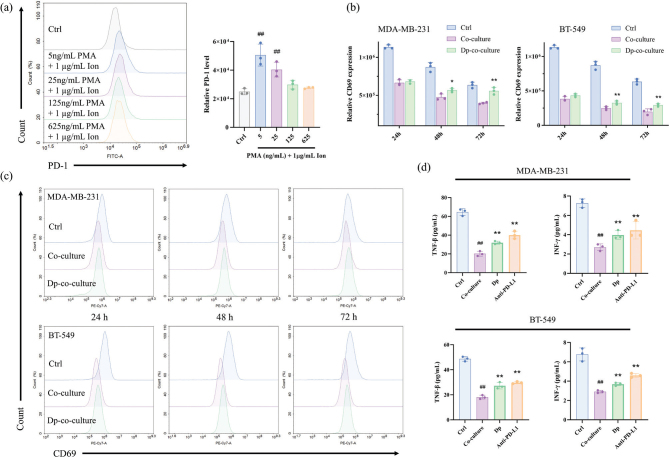
Delphinidin (Dp) reduces programmed cell death-ligand 1 (PD-L1) expression in exosomes from triple-negative breast cancer (TNBC) cells, thereby enhancing the reactivation of Jurkat cells. (a) Programmed death-1 (PD-1) expression was assessed via flow cytometry to determine the optimal conditions for Jurkat cell activation, which were found to be 5 ng/mL PMA and 1 μg/mL Ion. (b, c) Flow cytometry was used to analyze the expression of Cluster of Differentiation 69 in the co-culture system, where the control group involved activation of Jurkat cells alone with 5 ng/mL PMA and 1 μg/mL Ion. The co-culture group consisted of TNBC cells co-cultured with Jurkat cells, and the Dp-co-culture group involved co-culturing with TNBC cells treated with 80 μmol/L Dp. (d) After 48 h of co-culture, the levels of interferon-γ (IFN-γ) and tumor necrosis factor-β (TNF-β) were measured using enzyme-linked immunosorbent assay (ELISA) in the control, co-culture, Dp-co-culture, and atezolizumab programmed cell death-ligand 1 (Anti-PD-L1)-co-culture groups, and the Anti-PD-L1-co-culture group involved co-culturing with TNBC cells treated with 30 μg/mL atezolizumab. ## indicates *p* < 0.01 *vs.* control group. * indicates *p* < 0.05, ** indicates *p* < 0.01, *vs.* co-culture group.

This study examined whether Dp enhances Jurkat cell activity by reducing PD-L1 expression in exosomes derived from TNBC cells, thereby inhibiting TNBC progression. Flow cytometry results showed that PMA and Ion successfully activated Jurkat cells, increasing PD-1 expression. However, as PMA concentration increased, PD-1 mean fluorescence intensity decreased, with the highest PD-1 expression observed at 5 ng/mL PMA and 1 μg/mL Ion (Fig. 5a), which were selected for subsequent experiments.

Cluster of Differentiation 69, a C-type lectin receptor, serves as an early marker of T-cell activation. Flow cytometry was used to assess Cluster of Differentiation 69 expression on Jurkat cells in control, co-culture, and Dp-co-culture groups with TNBC cells. Cluster of Differentiation 69 expression significantly decreased in the co-culture group compared to the control group at 24, 48, and 72 h. However, in the Dp-co-culture group, Cluster of Differentiation 69 expression increased at 48 and 72 h compared to the co-culture group (Fig. 5b and c). These findings suggest that Dp restores Cluster of Differentiation 69 expression levels in a co-culture system.

An ELISA was employed to measure cytokines IFN-γ and TNF-β levels in control, co-culture, Dp-co-culture, and Anti-PD-L1-co-culture groups. In the control groups of MDA-MB-231 and BT-549 cells, IFN-γ and TNF-β levels were 7.26 pg/mL and 64.56 pg/mL, and 6.76 pg/mL and 48.70 pg/mL, respectively. After 48 h of co-culture with Jurkat cells, these cytokine levels significantly decreased. However, in the Dp-co-culture and Anti-PD-L1-co-culture groups, IFN-γ and TNF-β increased significantly (Fig. 5d). These results suggest that Dp enhances the secretion of IFN-γ and TNF-β by Jurkat cells, effectively reversing their immunosuppressive state in the co-culture system.

### Dp modulates PD-L1 expression by targeting the JAK2/STAT3 signaling pathway

**Fig. 6:**
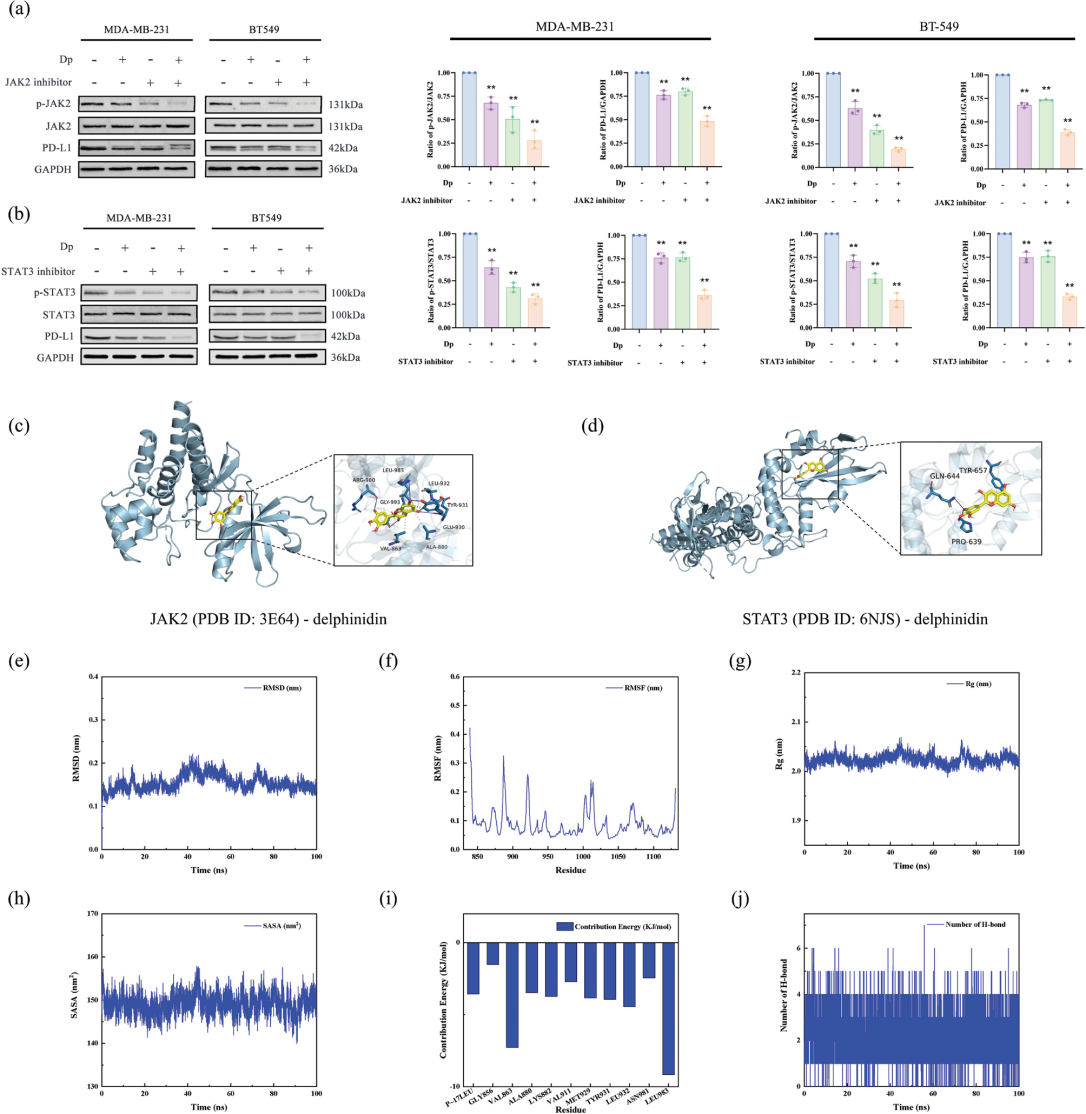
Delphinidin (Dp) modulates programmed cell death-ligand 1 (PD-L1) expression via the JAK2/STAT3 signaling pathway in triple-negative breast cancer (TNBC) cells. (a) Western blot analysis assessed the expression of p-JAK2, total JAK2, and PD-L1 in TNBC cells using a Dp concentration of 80 μmol/L and a JAK2 inhibitor concentration of 1 μmol/L. (b) Western blot analysis examined p-STAT3, total STAT3, and PD-L1 levels in TNBC cells, with Dp at 80 μmol/L and a STAT3 inhibitor concentration of 45 μmol/L. (c) Molecular docking simulations depicted the interaction between Dp and JAK2. (d) Molecular docking simulations illustrated the interaction between Dp and STAT3. Molecular dynamics simulations were conducted for JAK2 and Dp, and the (e) root mean square deviation (RMSD), (f) root mean square fluctuation (RMSF), (g) radius of gyration (Rg), (h) solvent accessible surface area (SASA), (i) Contribution energy and (j) H bonds were calculated separately. ** indicates *p* < 0.01, *vs.* co-culture group.

To further explore the potential molecular mechanisms by which Dp modulates PD-L1 expression in TNBC cells, we examined the JAK2/STAT3 signaling pathway, a key upstream regulator of PD-L1 expression. TNBC cells were treated with Dp alone or in combination with JAK2 and STAT3 inhibitors. Western blot analysis showed that Dp reduced the p-JAK2/JAK2 (Fig. 6a) and p-STAT3/STAT3 (Fig. 6b) ratios in both TNBC cell lines, leading to a concurrent downregulation of PD-L1 expression. This indicates that Dp inhibits JAK2 phosphorylation, thereby suppressing the phosphorylation and transcriptional activity of STAT3, ultimately reducing PD-L1 levels. Additionally, molecular docking simulations using AutoDock Vina 1.1.2 revealed that Dp binds to the active sites of JAK2 (PDB ID: 3E64) and STAT3 (PDB ID: 6NJS). The docking binding energy between JAK2 and Dp is −8.5 kcal/mol. Hydrophobic interactions with Dp are facilitated by residues VAL863, ALA880, TYR931, and LEU983, while hydrogen bonds are formed between Dp and residues GLU930, TYR931, LEU932, ARG980, GLY993, and LEU983 (Fig. 6c). The binding energy between STAT3 and Dp is −6.0 kcal/mol, with TYR657 participating in hydrophobic interactions and GLN644 and PRO639 forming hydrogen bonds with Dp (Fig. 6d).

To further validate the reliability of molecular docking results, we selected the upstream protein JAK2 with Dp for MD simulations. The analyses of Root Mean Square Deviation (RMSD), Root Mean Square Fluctuation (RMSF), Radius of Gyration (Rg), hydrogen bonds, and Solvent Accessible Surface Area (SASA) indicated that JAK2 remained relatively stable throughout the simulation (Fig. 6e–j). Using protein-ligand MM-PBSA analysis, the total binding free energy was calculated to be −99.291 kJ/mol. Certain residues, such as VAL863 and ALA880, significantly contributed to the binding energy, highlighting their crucial roles in molecular interactions (Fig. 6i). The number of hydrogen bonds varied between 2 and 4 during the simulation, suggesting that the intermolecular hydrogen bonds were relatively stable (Fig. 6j). These results suggest that Dp suppresses PD-L1 expression in TNBC cells via the JAK2/STAT3 signaling pathway, thereby inhibiting TNBC progression.

## Discussion

TNBC is marked by high recurrence and mortality rates, largely due to the lack of effective therapeutic targets. While chemotherapy remains a common treatment, available systemic options are limited and often lead to poor prognoses, as these agents indiscriminately affect both healthy and cancerous cells. Therefore, identifying natural anticancer agents that specifically target TNBC while minimizing harm to normal cells is crucial. Dp, an anthocyanin monomer abundant in many pigmented fruits and vegetables, is known for its numerous health benefits. Although the anti-cancer properties of Dp are well-documented, the complex molecular mechanisms underlying its effects require further investigation.

Numerous studies have demonstrated that Dp can inhibit cancer cell proliferation and migration. Yun et al. (20) used an Methyl Thiazolyl Tetrazolium assay to determine Dp’s impact on cell viability, revealing that Dp significantly inhibited the proliferation of human colon carcinoma HCT116 cells. Similarly, research by Kang et al. (21) showed that Dp reduced cell viability and inhibited migration in human osteosarcoma HOS and U2OS cells in a dose-dependent manner. In our study, we evaluated Dp’s effects on toxicity, proliferation, and migration of TNBC cells (MDA-MB-231 and BT-549) and normal breast epithelial cells (MCF-10A) using CCK-8, colony formation, and scratch assays, respectively. Results indicated that Dp inhibited the proliferation and migration of TNBC cells. The IC50 value for the normal MCF-10A cells was considerably higher than for TNBC cells, suggesting that Dp’s toxic effect on normal cells is less pronounced than on cancerous cells. These findings reinforce that Dp exerts anti-TNBC effects in a concentration-dependent manner, consistent with the results of Ozbay et al. (22).

PD-L1 is a type I transmembrane protein predominantly expressed on the plasma membrane. In the TME, tumor cells upregulate PD-L1 expression on their surfaces, which binds to PD-1 on tumor-specific CD8+ T cells. This interaction inhibits T-cell activation or induces apoptosis, resulting in reduced secretion of anti-tumor cytokines, such as IFN-γ and IL-2. Consequently, T-cells fail to detect tumor cells, allowing cancer cells to evade immune surveillance and impairing the host’s immune response (23). Additionally, TNBC has higher immunogenicity, more tumor-infiltrating lymphocytes, and increased PD-L1 expression compared to other breast cancer subtypes, making it more amenable to immune checkpoint blockade therapies (24). To assess PD-L1 expression, we performed Western blot analysis on both TNBC cells and MCF-10A cells. The results revealed that TNBC cells exhibit higher PD-L1 levels than MCF-10A cells, with MDA-MB-231 cells showing the highest levels, consistent with findings by Jing et al. (25). TNBC cells were treated with Dp, and PD-L1 expression was measured using Western blotting and flow cytometry. Both Dp and the PD-L1 inhibitor Anti-PD-L1 effectively reduced PD-L1 expression in TNBC cells, suggesting that Dp has a similar effect to Anti-PD-L1 in modulating PD-L1 levels.

Research highlighted the crucial role of exosomes in intercellular communication, particularly in transporting nucleic acids, proteins, and lipids between cells (26). Tumor-derived exosomes, in particular, carry active PD-L1, which can interact with PD-1 on T cells, suppressing the anti-tumor immune response (27). Theodoraki et al. (28) isolated exosomes from the serum of patients with squamous cell carcinoma of the head and neck, finding a correlation between exosomal PD-L1 levels and tumor grade. Similarly, Li et al. (29) demonstrated that in NSCLC patients, elevated exosomal PD-L1 levels were linked to larger tumors, distant metastases, and advanced TNM staging compared to healthy controls. In this context, we isolated exosomes from the TNBC cell line using ultracentrifugation and characterized them via TEM, NTA, and Western blotting, confirming successful extraction. PD-L1 expression in these exosomes was assessed using ELISA post-Dp treatment. The findings revealed that Dp reduced PD-L1 levels, suggesting that Dp may inhibit exosomal PD-L1 expression, thereby blocking immune evasion and exerting anti-TNBC effects. Our research demonstrates that Dp reduces PD-L1 expression on the surface of TNBC cells and in exosomes derived from them. However, the precise mechanisms underlying this effect remain unclear. The JAK2/STAT3 signaling pathway may play a role (30), and post-translational modifications of PD-L1 could influence its sorting into exosomes (31). Recent studies indicate that the knockout or inhibition of exosomal PD-L1 may serve as a therapeutic strategy to modify the immunosuppressive TME (9). These findings highlight the potential significance of exosomal PD-L1 in the treatment of TNBC.

T-cell activation is essential for orchestrating the immune response. Central to this process in Jurkat cells is cytokine production, which enhances T-cell proliferation and differentiation (32). Another indicator of T-cell activation is the upregulation of Cluster of Differentiation 69, a classic activation marker (33). These early events are critical for mounting an effective immune response. However, the PD-1/PD-L1 interaction inhibits T-cell receptor signaling, further restricting T-cell activation and promoting immune escape, which diminishes the efficacy of anticancer therapies and results in poor clinical outcomes. In this study, we used PMA and Ion to activate Jurkat cells, promoting IFN-γ and TNF-β secretion to mimic effector T cells. Transwell chambers were employed to establish a co-culture model of Jurkat and TNBC cells, simulating the TME in vitro. Jurkat cell immune activity was suppressed following interaction with TNBC cells. This co-culture system was used to evaluate the therapeutic effect of Dp on the immunosuppressive TME. The results showed that Dp treatment increased Cluster of Differentiation 69 expression and enhanced the secretion of IFN-γ and TNF-β in Jurkat cells within the co-culture system, suggesting that Dp may enhance the TME by restoring Jurkat cell activity. These findings align with those of Xie et al. (34), who demonstrated in a co-culture model of lung cancer cells and activated Jurkat cells that lapatinib decreased PD-L1 expression, upregulated Cluster of Differentiation 69 levels, and enhanced IFN-γ secretion in Jurkat cells.

The JAK2/STAT3 signaling pathway is crucial in cancer development, involved in processes like immune regulation, angiogenesis, cell proliferation, and differentiation. It is closely associated with tumor behaviors such as development, progression, metastasis, and invasion, and its aberrant expression holds prognostic significance (35, 36). Previous studies have demonstrated that the JAK2/STAT3 pathway regulates PD-L1 expression in various cancers, including head and neck squamous, lung, gastric, and prostate cancers (37, 38). Jha et al. (39) found that JAK2/STAT3 activation supports PD-L1 expression, promoting survival, invasion, metastasis, and chemoresistance in oral squamous cell carcinoma. In our study, TNBC cells were treated with Dp alone or in combination with JAK2 and STAT3 inhibitors. Western blot analysis showed that Dp inhibited the phosphorylation of JAK2 and STAT3 and reduced PD-L1 expression, with a more pronounced effect when combined with the inhibitor. Furthermore, molecular docking and MD simulations confirmed that Dp interacts with JAK2 and STAT3, supporting the hypothesis that Dp reduces PD-L1 and levels via the JAK2/STAT3 signaling pathway, thereby exerting anti-TNBC effects.

**Fig. 7:**
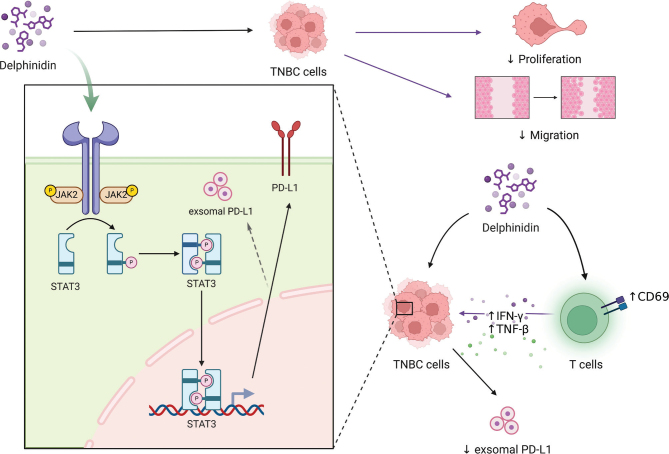
Delphinidins’ (Dp) modulation of the Janus Kinase 2 (JAK2)/Signal Transducer and Activator of Transcription 3 (STAT3) pathway inhibits programmed cell death-ligand 1 (PD-L1) expression and reinstates T-cell activity in triple-negative breast cancer.

In this study, we demonstrated the role of Dp in inhibiting TNBC cell proliferation and migration, reducing PD-L1 expression in both TNBC cells and their derived exosomes, restoring Jurkat cell activity, and ameliorating the immunosuppressive TME. Furthermore, we confirmed that Dp may downregulate PD-L1 expression by inhibiting the phosphorylation of the JAK2/STAT3 signaling pathway, thereby impeding TNBC progression. These findings provide new insights into the therapeutic potential of Dp for targeting TNBC (Fig 7).

## Conclusion

The study suggests that Dp can inhibit TNBC growth by targeting the JAK2/STAT3/PD-L1 pathway, supporting the chemopreventive potential of anthocyanins against TNBC.
